# A Comprehensive Study on Folate-Targeted Mesoporous Silica Nanoparticles Loaded with 5-Fluorouracil for the Enhanced Treatment of Gynecological Cancers

**DOI:** 10.3390/jfb15030074

**Published:** 2024-03-20

**Authors:** Aliyah Almomen, Adel Alhowyan

**Affiliations:** 1 Department of Pharmaceutical Chemistry, College of Pharmacy, King Saud University, P.O. Box 22452, Riyadh 11495, Saudi Arabia; 2 Department of Pharmaceutics, College of Pharmacy, King Saud University, Riyadh 11495, Saudi Arabia; adel-ali@ksu.edu.sa

**Keywords:** mesoporous silica, nanoparticle, 5-fluorouracil, folic acid, in-vitro, cytotoxicity

## Abstract

Background: Gynecological cancers are a significant public health concern, accounting for 40% of all cancer incidence and 30% of deaths in women. 5-Fluorouracil (5-FU) can be used with chemotherapy to improve treatment in advanced-stage gynecological cancer. Mesoporous silica nanoparticles (MSNs) can improve drug effectiveness and reduce toxicity. Folic acid can target folate receptors in epithelial malignancies like ovarian and cervical cancer. Methods: The mixture of MSN-NH2 was synthesized by dissolving N-lauroylsarcosine sodium in a water–ethanol mixture, adding APTES and TEOS, and heating at 80 °C for 18 h, before being fully characterized. The drug is loaded into a 5-FU solution and functionalized with folate. The drug release mechanism, as well as ex vivo intestinal permeation from MSN-NH2 formulations, was tested. The cell viability study of the nanoparticles was evaluated in various cancer cell lines, and the cellular uptake was measured indirectly using HPLC. Results: The study analyzed the amine content, propylamine loading, and drug loading capacity of MSN-NH2 nanoparticles. It found that the loading of propylamine was around 0.733 mmol/g, and the surface density was 0.81 molecules/nm. The study also showed that the surface decoration of MSN-NH2 with folic acid was successfully achieved. The release rate of 5-FU from MSN-NH2 was slow and controlled, with a slower rate at pH 5.5. The study found that the amin surface functionalization of MSN-NH2 nanoparticles can reduce potential toxicity in ovarian and cervical cancer cells. Conclusions: Based on the results, the encapsulation of 5-FU and functionalization of MSN-NH2 with folic acid can serve as potential carriers for 5-FU in treating gynecological cancer.

## 1. Introduction

Gynecological cancers such as uterine, ovarian, and cervical cancers are severe public health hazards since they continue to be a leading cause of cancer-related mortality [1]. Around 40% of all cancer cases and over 30% of all cancer-related deaths in women globally are believed to be gynecological malignancies, with an estimated yearly incidence of over 3.6 million cases and a mortality rate exceeding 1.3 million [1].

Cervical cancer (CC) is the fourth most common cancer among women globally. In 2020, 604,000 new cases of CC were diagnosed, and 342,000 deaths were reported, with 90% of cases occurring in low- and middle-income countries [1]. CC is caused by specific forms of human papillomavirus (HPV) and can be avoided by getting vaccinated against HPV, getting frequent cervical cancer screenings, and treating precancerous lesions known as cervical intraepithelial neoplasia [2]. Access to these therapies, however, varies significantly across the globe, resulting in considerable differences in cervical cancer incidence and fatality rates [2]. Chemotherapy is a treatment option for certain CC stages, often combined with radiation therapy and surgery. The specific drugs and timing depend on the cancer stage, patient health, and healthcare team’s treatment plan. Early-stage CC may not require chemotherapy, while locally advanced CC (stage IB2–IVA) may require chemoradiation [3]. Cisplatin, paclitaxel, and 5-FU are standard chemotherapies used in treating CC [3].

Ovarian cancer is another gynecological severe disease that affects women of all ages [4,5]. In 2020, 313,959 new cases were diagnosed globally, with 207,252 deaths, making it the third most common type of gynecological cancer in women worldwide [4]. It is more common in older women, with cases in older women comprising half of all cases occurring in women. Women with a family history of ovarian or breast cancer may be at higher risk. Early detection is difficult, leading to a 5-year relative survival rate of 50% for all stages combined [6]. Early diagnosis and treatment are more effective, with a 92% survival rate for localized ovarian cancer [7]. Treatment options include surgery, chemotherapy, targeted therapy, or a combination of these approaches, depending on the cancer stage [8].

5-Fluorouracil (5-FU) is an anticancer used to treat cervical and ovarian cancer; however, it is not a first-line treatment choice [9]. In advanced-stage cervical cancer, it can be used with other chemotherapy medications like carboplatin to improve results [9]. It can also operate as a radiosensitizer, improving the efficacy of radiation treatment [10]. It may also be utilized as a second-line or later treatment option in ovarian cancer, particularly in conjunction with other chemotherapy medicines such as cisplatin, paclitaxel, or carboplatin [11]. However, the use of 5-FU in ovarian cancer may cause nausea, vomiting, exhaustion, hair loss, and an increased risk of infection.

Optimum drug delivery systems can improve drug effectiveness, decrease toxicity, and help overcome drug resistance [12]. Controlled drug release systems receive more attention; these can reduce the toxic effect of the medication by controlling the drug release at the site of the diseases [13]. Mesoporous silica nanoparticles (MSNs) have been used as a carrier for drug delivery, cellular imaging, and photodynamic therapy. MSNs are promising drug delivery systems that can be tailored as stimulus-responsive carriers due to their excellent physicochemical properties [14,15]. The surface modification of MSNs, such as the formation of aminofunctionalized mesoporous silica nanoparticles (MSN-NH2), can further enhance the encapsulation and loading of active agents inside the nanoparticles. Different mechanisms were suggested to explain the enhanced loading of the drug inside the MSN-NH2, such as hydrogen bonding and electrostatic interactions [14,15]. Also, the surface of MSN-NH2 can be easily conjugated with a targeting agent or responsive polymer to enhance the localization of the drug at the site of interest. The conjugation of the targeting agent on the surface of MSN-NH2 would enhance the biological membrane permeation of the carrier and can reduce drug resistance [16,17]. The advantages of MSNs as a drug delivery system include their high surface area and pore volume, which increase the encapsulation of drugs, as well as their adjustable pore size, structural stability, easy conjugation procedure, and biocompatibility [18]. These advantages of MSNs can be used to design and tailor an optimum drug delivery system. These parameters affect the cellular uptake, transport, toxicity, and pharmacokinetic fate of the drug delivery system [19].

Generally, nanocarriers can be integrated for targeting by conjugation with targeting agent such as folate. The functionalization of MSN is widely explored to improve the specificity of chemotherapy, which would reduce the drug’s toxicity. Similar to other nanocarriers, MSNs can accumulate in the malignant tumor via the enhanced permeability and retention (EPR) effect [20]. Active targeting can increase the uptake of the nanocarrier by cancer cells through receptor-mediated endocytosis. Some receptors are overexpressed on malignant cells such as folate receptors and folic acid can be attached to the surface of the MSNs to enhance the drug delivery to cancer cells [21]. Folic acid complements the folate receptors that are upregulated in some cancer cells. A study conducted by Ma et al. [22] for photodynamic therapy used the functionalization of the MSN-NH2 with folate to deliver 5-aminolevulinic acid to work against B16F10 skin cancer cells, and the nanoparticles showed high cytotoxicity to the cancer cells. Another study showed the utilization of amine functionalization on the surface of MSN and covalent binding of the folate to enhance the cellular uptake and targeting of docetaxel in breast cancer cells. The study demonstrated that the cellular uptake and apoptosis of the folate-conjugated MSN-NH2 loaded with docetaxel were higher than those of MSN-NH_2_-docetaxel [23]. Functionalizing the nanoparticle surface with some targeting ligand would avoid P-glycoprotein (P-gp)-mediated drug resistance by avoiding its recognition and facilitating receptor-dependent endocytosis of the nanocarrier, hence increasing the intracellular drug uptake [24]. Liong et al. [25] showed that functionalized MSN with folate loading and the encapsulation of camptothecin enhanced cytotoxicity to cause around 60% cell death after 24 h for pancreatic cancer cell line, in comparison to non-targeted nanoparticles, which showed a cytotoxicity of around 30%.

However, the physicochemical properties of folic-acid-conjugated nanocarriers should be designed using biocompatible linkers with low toxicity profiles to obtain the desired activity while keeping the drug delivery system intact until it reaches the active site [18]. The use of linkers and spacers is essential for the integrity of the drug carrier and they are used to conjugate the carrier to the targeting agents, as well as to facilitate the drug release at the target site. Linkers carrying modifiable groups such as thioether, acetal, carbamate, amine, hydroxyl, and carbodiimide, can facilitate the conjugation with the targeting agent and enable the release of the drug from the nanocarrier [18]. The ethyl-3-(3-dimethylaminopropyl)carbodiimide (EDC) coupling reaction is commonly used to conjugate the carboxylic acid group with primary amine groups, leading to amide bond formation [18]. For example, aminopropyl contains an amino group and can therefore serve as a linker on the MSN surface. The folate MSN conjugate can be achieved by the formation of an amide bond between the amino group of MSN-NH_2_ and the folic acid carboxyl group. Surface functionalization such as covalent bond formation between folic acid and a nanocarrier utilizing stable chemical linkers such as amide bonds would verify that the targeting agents remain attached and prevent early detachment before the active site is reached [18]. Selvaraj et al. used gold nanorods centralized in MSN and functionalized with folate as a diagnostic agent [26]. The system showed high specificity in tumors at low doses in vivo. Also, they highlighted the effect that surface characteristics have on carrier-induced hemolysis. They suggested that when using 3-aminopropyl triethoxysilane as a linker, a low hemolytic effect was observed, in comparison to the unfunctionalization, which showed a significantly higher level of hemolysis [26].

Various disease-specific ligand–receptor pairs have been identified, such as folic acid, peptides, antibody fragments, and whole antibodies [17,27]. Due to their selectivity and specificity to specific cell receptors, those molecules increase nanoparticle selectivity and specificity [27]. Folic acid is used as a targeting moiety for folate receptors, which are overexpressed in some epithelial cancers, such as ovarian, cervical, colorectal, and breast cancer cells, relative to many types of normal cells, making folate conjugates an attractive tool for use in cancer-targeted therapy [17]. In ovarian cancer, for example, the folate receptor alpha FRα is highly expressed (72% in primary tumors and 81.5% in recurrent tumors), making it a potential target for folate-targeted chemotherapy [28]. FRα is also overexpressed in cervical cancer [29]. The overexpression of FRα has been linked to more aggressive types of cervical cancer and a worse prognosis [29]. Some studies have shown that folate-targeted anticancer nanoparticle systems would be beneficial in patients with cervical or ovarian cancer who overexpress folate receptors [3,4].

In this work, we prepared amino-functionalized silica nanoparticles using a novel synthesis scheme, as described in our previous publications [30,31]. We then used a simple method for the conjugation of the folate on the surface of MSN-NH2 using EDC in aqueous media without using N-hydroxysuccinimide. Finally, the application of these formulations to the viability of ovarian and cervical cancer was conducted. To our knowledge, not much work has been performed using folate f-functionalized MSN-NH2 loaded with 5-FU on ovarian and cervical cancer. Table 1 summarizes the literature on the utilization of different types of nanoparticles loaded with 5-FU for use in cancer treatment.

## 2. Materials and Methods

### 2.1. Material

N-lauroylsarcosine sodium, tetraethyl orthosilicate (TEOS), 3-aminopropyltriethoxysilane (APTES), 5-fluorouracil, and folic acid were acquired from Sigma-Aldrich Chemical Co. (St Louis, MO, USA).

### 2.2. Preparation of Aminofunctionalized Silica Nanoparticles (MSN-NH2)

MSN-NH2 was synthesized in situ, as described in our previous publication [30]. In brief, 1.4667 g of N-lauroylsarcosine sodium was dissolved in 33 mL of a 10:1 water–ethanol solution. After that, 4 mL of 0.1 N HCl was added, and the mixture was stirred for a full h. Following the addition of approximately 150 µL of aminofunctionalizing agents (APTES), the mixture was agitated for 10 min. After that, 1.5 mL of TEOS was added, and the mixture was shaken for an additional 10 min. After the mixture was sonicated using an ultrasonic water bath, it was given a 1 h rest period. Thereafter, the mixture was heated at 80 °C for 18 h. Ultimately, the particles were extracted using ultracentrifugation, followed by three rounds of washing in deionized water and a 12 h drying period at 60 °C in the oven. The solvent extraction method was used to remove the surfactant molecules. In a nutshell, the particles were mixed with 8.01 g of ammonium acetate in a 4:1 ethanol to water solution mixture and refluxed for 12 h at 90 °C. After that, the nanoparticles were similarly recovered by ultracentrifugation, cleaned three times with deionized water, and dried for 12 h at 60 °C in an oven [46].

### 2.3. Estimation of Amine Content by CHN and TGA Analysis

#### 2.3.1. Carbon, Hydrogen, and Nitrogen Elemental Analysis (CHN Analysis)

To calculate the amino content, the sample was analyzed using PerkinElmer CHNS/O analyzer 2400 (Perkin Elmer, Shelton, CT, USA). Blank, K-factor, and standard (Acetanilide) runs were carried out to stabilize the machine before starting sample analysis.

#### 2.3.2. Thermogravimetric Analyses (TGA)

Using a differential scanning calorimeter (TGA-DSC; SDT Q600 V20.9 Build 20, TA Instruments, Champaign, IL, USA), TGA analyses were carried out to determine the amount of propylamine in the particles. The heating rate and the nitrogen flow were 5 °C/min and were 50 mL/min, respectively.

### 2.4. Drug Loading and Preparation of MSN-NH2 Loaded 5-FU (MSN-NH2-5FU)

Drug loading was performed by passive loading, in which MSN-NH2 (25 mg) was added to 5-FU (1 mg/mL) solution in PBS. The mixture was sonicated by probe sonication (Badnelin, Berlin, Germany) for 5 min and then stirred overnight. The nanoparticles were recovered by centrifugation at 6000 rpm for 1 h and then lyophilized for 24 h. The amount of the loaded drug was measured indirectly by analyzing the amount of the drug in the supernatant using high-performance liquid chromatography (HPLC) [35,47]. Encapsulation efficiency (EE%) and loading capacity (LC%) were calculated by the following equations:

(1) $$EE\;\left(\%\right)=\frac{W\;total-W\;free}{W\;total}\times 100$$

(2) $$LC\;\left(\%\right)=\frac{W\;total-W\;free}{W}\times 100$$

where W total is the initial weight of 5-FU before loading, W free is the unloaded amount of the drug in the solution, and W is the total weight of the formulation [14].

### 2.5. Functionalization of 5-FU-Loaded MSN-NH2 with Folate (MSN-NH2-5FU-FA)

A total of 45 mg of 1-ethyl-3-(3 dimethyl aminopropyl) carbodiimide (EDAC) aqueous solution was added to 60 mg of 5-FU-loaded MSN-NH2 at 1 mg/mL, and the mixture was stirred at 25 °C for 30 min. Then, an excess of folic acid (60 mg) was added, and the reaction between folic acid and the amino surface proceeded for 3 h. The loaded MSN-NH2 functionalized by folate was separated by high-speed centrifugation at 6000 rpm. Finally, unreacted folic acid was removed by washing the nanoparticles with DMSO (60 mL), then with distilled water (60 mL), before being lyophilized for 24 h [48]. To quantify the amount of 5-FU in the folate-functionalized MSN-NH2, the supernatants, and the washed solutions were collected, and the residual 5-FU amount was measured by HPLC. EE% and LC% were calculated according to the equation mentioned previously [49]. LC% was further confirmed and calculated by extracting the drug using the following procedure. The loaded MSN-NH2-5FU-FA or MSN-NH2-5FU nanoparticles were extracted using PBS (pH 7.4). Accurately weighed nanoparticles (5 mg) were dispersed in PBS (10 mL) and subjected to probe sonication for 15 min at 80% power. Then, the nanoparticle mixture was placed in the water bath for three days at 37 °C. The amount of 5-FU loaded in the nanoparticles was analyzed using HPLC (Agilent, Santa Clara, CA, USA) for 5-FU.

### 2.6. Particle Size, Particle Size Distribution, and Zeta-Potential Analysis

The Zetasizer Nanoseries-ZS (Malvern Instruments, Malvern, UK) was utilized to estimate the particle size, zeta potential, and polydispersity index (PDI) of the MSN. Following the use of an appropriate aqueous dilution to achieve a nanoparticle concentration of 100 μg/mL, the size distribution, PDI, and particle size of MSN were examined at 25 °C using the dynamic light scattering (DLS) mode [16,17]. After the MSN-NH2 was appropriately diluted in PBS or phosphate buffer pH (5.5) at room temperature, the zeta potential (mV) of the sample was measured using the instrument’s Laser Doppler Velocimetry (LDV) mode [50].

### 2.7. Particle Morphology by Scanning Electron Microscopy (SEM) and Transmission Electron Microscope (TEM)

Micrographs of the samples were obtained using scanning electron microscopy (SEM) (Zeiss EVO LS10; Cambridge, UK) using the gold sputter technique. Using a Q150R Sputter unit from Quorum Technologies Ltd. (Lewes, East Sussex, UK), the MSN-NH2 formulations were vacuum-dried and coated with gold for 60 s at 20 mA in an argon atmosphere. For the micrographs, the zone magnification was maintained between 10,000 and 15,000×. Investigations were carried out at 1 and 15 kV [16]. The morphology of MSN formulations and particle size were also determined by TEM (JEM-1011, JEOL, Tokyo, Japan) at 60 kV. Also, the TEM (JEM-1011, JEOL, Japan) was utilized to determine the particle size and morphology of MSN formulations at a voltage of 60 kV.

### 2.8. Fourier Transform Infrared Spectroscopy (FTIR)

FTIR (Perkin Elmer spectrum BX, Perkin Elmer, Shelton, CT, USA) was used to compare the infrared spectra of the drug and MSNs. The materials were prepared as potassium bromide (KBr) pellets, and spectra were collected across 4400–350 cm^−1^ wavenumbers using three scans and a 2 cm^−1^ resolution [23,51,52,53].

### 2.9. Differential Scanning Calorimetry (DCS)

DCS (DSC-8000 Perkin Elmer Instruments, Waltham, MA, USA) was used to measure the samples’ thermal characteristics. Over a temperature range of 25–350 °C, the samples were scanned at a scan rate of 10 °C/min.

### 2.10. The Proton Nuclear Magnetic Resonance (^1^H-NMR)

The ^1^H-NMR spectrum was obtained from a Bruker AV 300 spectrometer equipped with a 7T wide bore superconducting magnet. The samples contained 10 mg/mL sodium hydroxide, and the powder concentration was 20 mg/mL, dispersed in D_2_O. Chemical structures were illustrated using the CS ChemDraw^®^ Std 14 program.

### 2.11. In Vitro Drug Release

A dialysis membrane (molecular weight cut of 12 kD) was used to perform the in vitro release of MSN-NH2-5FU and MSN-NH2-5FU-FA. A weighed portion of MSN-NH2-5FU or MSN-NH2-5FU-FA, equal to one milligram of 5-FU, was mixed with one milliliter of water. The solution was placed in a dialysis membrane. The dialysis membrane was transferred to a 50 mL beaker containing 40 mL PBS (pH 7.4) or phosphate buffer (pH 5.5). The outer phase was stirred continuously at 100 rpm and maintained at 37 °C during the experiment. The sample was withdrawn and replenished at predetermined intervals with the same amount of receptor fluid. The percentage of the cumulative amount released for 5-FU was measured by HPLC [23,54].

Five kinetic models were used to fit the release data to evaluate the mechanism of the drug release. Drug release parameters were evaluated using the following models: zero-order, first-order, Higuchi, Korsmeyer–Peppas, and Hixon Crowell.

Zero-order equation: Q_t_ = Q_0_/K_0_t (3)

First-order equation: log Q_t_ = logQ_0_/K_1_t/2:303 (4)

Higuchi equation: Q_t_ = K_h_ t^1/2^ (5)

Korsmeyer–Peppas equation: Q_t_/Q_∞_ = K_p_t^n^ (6)

Hixon crowell: Q_0_^1/3^ − Q_t_^1/3^ = K·t (7)

In this case, Q_∞_, Q_0_, and Q_t_ stand for the total amount of the drug in nanoparticles, the initial amount of 5-FU, and the cumulative amount of drug released at time t, respectively. The zero-order release rate constant is denoted by K_0_. The amount of the drug was computed by plotting Q_t_ against time. The first-order release rate constant, denoted by K_1_, is determined by graphing the log (Q_t_/Q_1_) against time. After graphing Q_t_ against the square root of time, one can determine the Higuchi release rate constant, or K_h_. The constant n is the release exponent that is used to describe the various release mechanisms, and K_p_ stands for the release rate constant of the Korsmeyer–Peppas model. By graphing the log (Q_t_/Q_1_) against the log time, parameters K_p_ and n were determined. In the Hixon–Crowell model, the cube root of the percentage of remaining drug vs. time is correlated with the release from systems with the polymer erosion/dissolution, resulting in a change in the surface area and diameter of polymeric drug carrier beads or tablets. Q_t_ is the amount of drug left in the carrier at time t, Q_0_ is the amount of drug initially present in the formulation, and K is the rate constant for the Hixson–Crowell rate equation. These values were used for the model. Only the initial 60% of 5-FU released from the nanoparticles is covered by all of the utilized mathematical models [55].

### 2.12. Ex Vivo Intestinal Permeation

All studies were performed according to the Guidelines of the Animal Ethical Committee of King Saud University, Riyadh, Saudi Arabia, protocol number SE-19-151, and were approved on 13 February 2020. After sacrificing male Wistar rats weighing 200–250 g, the small intestine was excised and placed into Krebs buffer solution. The intestine was cut to accurately measure segments and rinsed with ice-cold Krebs buffer to remove luminal content. MSN-NH2-5FU-FU, MSN-NH2-5FU-FA-FU-FA, or a drug solution containing 250 μg of 5-FU was placed in the lumen of the intestine and tied on both sides. Then, each intestinal segment was placed in 10 mL of Krebs solution at 37 ± 0.5 °C with continuous aeration. Samples were taken from the receptor chamber at predetermined time intervals and replaced with an equal volume of Krebs solution. Finally, samples were diluted with water and were assayed for drug content using HPLC. Apparent permeability coefficient (Papp) can be calculated using Equation (8).

Papp = dQ/(dt × A × Co)(8)

where A is the area of the tissue (cm^2^), dQ/dt is the steady-state appearance rate on the acceptor side of the tissue, and Co is the initial concentration of the drug in the donor compartment [56].

### 2.13. In Vitro Cytotoxicity Assay

The cytotoxicities of 5-FU, MSN-NH2, MSN-NH2-5FU, MSN-NH2-5FU-FA and folic acid were evaluated in ovarian adenocarcinoma (SKOV-3) (ATCC^®^ HTB-77™), HPV18 positive cervical adenocarcinoma (HeLa) (ATCC^®^CRM-CCL-2™), metastatic cervical carcinoma HPV 16 and 18-positive cells (Ca Ski) ATCC^®^CRL-1550™), and HPV-negative cervical carcinoma cells (C 33-A) (ATCC^®^HTB-31™) cells. All cells were obtained from an American-type culture collection (ATCC, Manassas, VA, USA).

The cells were incubated at a temperature of 37 °C and a carbon dioxide concentration of 5%. The cell culture was created following the guidelines provided by ATCC. To conduct the cytotoxicity assay, cells were placed in a 96-well culture plate at a density of 1 × 10^4^ cells per well in 100 μL of culture media. The cells were then incubated for 24 h. Subsequently, the cells were subjected to a sequential dilution of the nanoparticles and the administration of 5-FU and FA in quantities equivalent to those in the nanoparticle system. The cells were subsequently incubated for 72 h. Following this, the cells were washed, and a solution containing 20 μL of 2.5 mg/mL of 3-(4,5-dimethylthiazol-2-yl)-2,5-diphenyltetrazolium bromide (MTT) in phosphate-buffered saline (PBS) was introduced to the cells. The cells were subsequently incubated for 4 h at 37 °C. The MTT solution was subsequently eliminated, and 100 μL of dimethyl sulfoxide (DMSO) was introduced to dissolve the formazan crystals. The absorbance at a wavelength of 540 nm was determined using a Spectramax 250 microplate reader manufactured by Molecular Devices, located in San Jose, CA, USA. Cell viability was calculated by dividing treated cells’ optical density (OD) by the OD of control cells and expressing the result as a percentage [57].

### 2.14. Intracellular Uptake

Cervical cancer and SKOV-3 cell lines were cultured in Dulbecco’s Modified Eagle Medium (DMEM), which was depleted of fetal bovine serum (FBS) for one week. The cells were then seeded at a density of 1 × 10^5^ cells per well in a 12-well plate and incubated for 24 h. Subsequently, the cells were treated with folate at 0.2 µg. Additionally, nanoparticles were introduced to the wells at a final concentration of 50 µg/mL in each experimental group. The cells were cultured for 1, 2, 4, and 8 h. Following that, the cells underwent three rinses with phosphate-buffered saline (PBS), were subjected to digestion with 0.25% trypsin, and subsequently underwent centrifugation at a force of 2000 times the acceleration due to gravity (2000× *g*). A total of 100 µL of methanol was introduced into the cell suspension, subjecting the cell mixture to multiple freeze–thaw cycles. The concentration of the NP in each sample was determined using High-Performance Liquid Chromatography (HPLC) [47].

### 2.15. Cellular Uptake Using Inductively Coupled Plasma Mass Spectrometry (ICP-MS)

SKOV-3 and Ca-ski, C-33a, and HeLa cells were treated with 0.06 μg/mL 5-FU, 15.6 μg/mL MSN-NH2-5-FU, or MSN-NH2-5-FU-FA. The cells were gently washed with PBS and 250 μL of 0.25% trypsin solution. After detachment, cells were centrifuged at 600× *g* and washed thoroughly with PBS. After removing the PBS, the pellets were dissolved in 70% nitric acid overnight and diluted to 2% before being analyzed using ICP-MS. 5-FU was quantified using CE- and ICP-MS with an internal standard of ^15^Sb and a detection limit of 6 ppb.

### 2.16. Statistical Analysis

The statistical analysis was carried out using GraphPad Prism software 8.0.1 (GraphPad Software Inc., San Diego, CA, USA). Except where otherwise stated, all experiments were conducted in triplicate. The results are expressed as mean ± SED, with a significance level of *p* < 0.05. When comparing three or more treatment groups, we used analysis of variance (ANOVA) and Tukey’s multiple comparisons test.

## 3. Results and Discussion

### 3.1. Estimation of Amine Content by CHN and TGA Analysis

#### 3.1.1. CHN Analysis

The following equations were used to calculate the amount of propylamine and the density of MSN-NH2 particles.

First, the δ_APTES_ in MSN-NH2 was calculated, starting from with amount of N, which was determined by CHN analyses (1.54%w). The moles of APTES in 1 g of MSN-NH2 were calculated according to the following Equation:

*n*_APTES_ = *n*_N_ = 1.54 × 10^−2^ g/14 g/mol = 0.733 mmol (9)

where 14 g/mol is the molar mass of N.

The weight percentage of Si within the MSN-NH2 was calculated as follows:

Si% = (*n*_N_ mol × 28 g/mol) × 100 = 2.04%w (10)

where 28 g/mol is the molar mass of Si.

The amount of oxide in 1 g of MSN-NH2 was calculated as follows:

*m* = 1 g − (6.99 × 10^−2^ g + 1.59 × 10^−2^ g + 1.54 × 10^−2^ g + 2.044 × 10^−2^ g) = 0.88 g (11)

where 6.99 × 10^−2^ g, 1.59 × 10^−2^ g, 1.54 × 10^−2^ g, and 2.044 × 10^−2^ g are, respectively, the masses of C, H, N, and Si in 1 g of MSN-NH2.

Thus, by considering the specific surface area (SSA) of MSN-NH2 powder (619.14 m^2^/g), the surface density of MSN-NH2, δ_APTES_, in the MSN-NH2 was determined using the following equation:

δ_APTES_ = (*n*_APTES_ (mol) × NA/SSA_MSN-NH2_ × *m*) × 10^−18^ m^2^/nm^2^ = 0.81 molecules/nm^2^ around 1 molecules/nm^2^

where NA is the Avogadro’s number (6.022 × 10^23^ molecules/mol).

#### 3.1.2. From TGA Analyses

The moles of APTES in 1 g of MSN-NH2 were obtained from the 9% weight loss starting at 280 °C in TGA curves, according to the following equation:

*n*_APTES_ = *n*_N_ = 9 × 10^−2^ g/88 g/mol = 1.02 mmol (12)

where 88 g/mol is the molecular weight of the MSN-NH2 alkyl chain (C_5_H_14_N). It should be noted that the two ethoxide groups of the MSN-NH2 molecule were considered to be completely hydrolyzed on the grounds of CHN analyses.

The mass of oxide present in 1 g of MSN-NH2 was calculated as follows:

*m* = 1 g − (*n*_APTES_ (mol) × 163.248 g/mol + 0.01 g H_2_O) = 0.823 g (13)

where 163.248 g/mol is the molecular weight of the attached APTES (C_5_H_14_NSiO_3_), assuming both ethoxide groups were lost. Thus, by considering the specific surface area (SSA) of the adopted MSN-NH2 (619.14 m^2^/g), NA is Avogadro’s number (6.022 × 10^23^ molecules/mol). The surface density of APTES, δ_APTES_, in the MSN-NH2 sample was calculated using the following equation:

δ_APTES_ = (*n*_APTES_ (moles) × NA/SSA_MSN-NH2_ × *m*) × 10^−18^ m^2^/nm^2^ = 1 molecules/nm^2^. (14)

**Figure 1:**
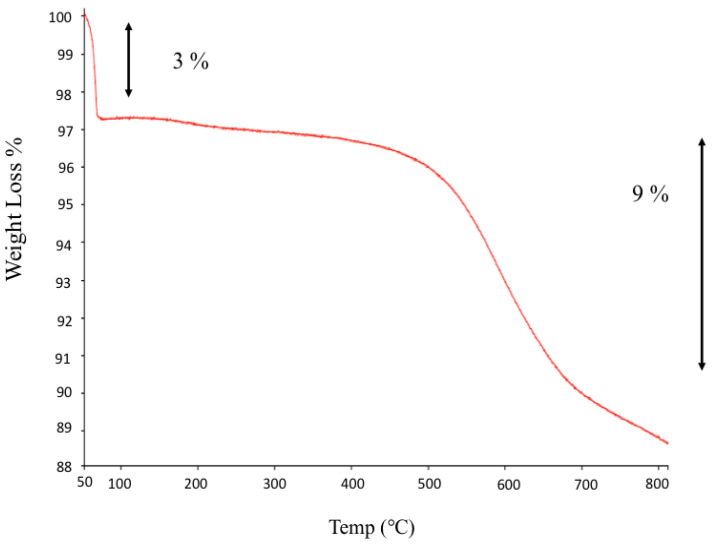
The TGA of MSN-NH2.

The loading of propylamine on the MSN-NH2 was determined from CHN elemental analysis and TGA. The details of the analysis data were as follows: carbon (6.99%), hydrogen (1.59%), and nitrogen (1.54%). This suggests that the loading of propylamine in MSN-NH2, according to the calculations, is around 0.733 mmol/g [58]. The surface density was calculated at approximately 0.81 molecules/nm^2^. The C/N molar ratio of MSN-NH2 is 5.2, close to the hypothetical value for an APTES residue that lost two ethoxy groups, at 4.97. The TGA analysis showed a weight loss of about 12% at up to 800 °C (Figure 1). Below 100 °C, around 3% weight loss occurs, due to the loss of surface hydration. At a higher temperature range, the TG curve of MSN-NH2 showed a marked weight loss (around 9%). This weight loss could be due to the decomposition of the alkyl groups of propylamine molecules on the surface of the MSN-NH2 [58]. According to the calculation from TGA, the loading of propylamine was in good agreement and close to the value calculated from CHN analysis, which is 1.02 mmol/g. The calculated surface density is also close to the CHN analysis value, 1.2 molecules/nm^2^ [58].

### 3.2. Nanoparticles DL%, EE% and Characterizations

The loading of 5-FU by MSN-NH2 was carried out in PBS at pH 7.4 (Table 2). At an acidic pH, 5-FU is protonated, which reduces the negative charge of 5-FU. As pH increases, the 5-FU loading capacity in MSN-NH2 increases and a maximum drug loading capacity of 15.26 ± 3.13% was attained at pH 7.4. MSN-NH2 is positively charged at a more comprehensive pH range because of the protonation of the amine groups. The charge on the 5-FU differs depending on the pH of the media. The deprotonation of 5-FU can occur at N1 or N3 or both to form the monoanion and dianion under alkaline pH conditions of between 7 and 10. When pH increases to 7.4, the deprotonation of 5-FU is enhanced, thereby increasing the electrostatic interaction between the positive charge of MSN-NH2 and the negative charge of 5-FU. Most 5-FU molecules in the solution transfer to monoanion and dianion, which generates negative charges and guides more 5-FU to become entrapped in the MSN [59,60]. The LC% of folate-functionalized 5-FU-loaded MSN-NH2 (MSN-NH2-5FU-FA) was 5.89 ± 0.32%, and this can be attributed to the washing step of unreacted folic acid from MSN-NH2-5FU-FA.

MSN-NH2 formulations have a positive charge in acidic and neutral aqueous solutions (Table 2). The ionization of the MSN-NH2-5FU formulation produces more stable nanoparticles with a zeta potential of about +30.4 ± 5.71 mV in PBS. In the folate-functionalized formulation (MSN-NH2-5FU-FA), the zeta potential was 8.57 ± 3.48 in PBS, which suggests successful functionalization on the surface of MSN-NH2-5FU. At a slightly acidic pH of 5.5, the zeta potential of MSN-NH2-5FU was decreased to 18 ± 2.9, which suggests a decrease in the total positive charge of MSN-NH2-5FU. The ionization of 5-FU in PBS may create an acidic microenvironment around MSN-NH2, which increases the positive zeta potential of MSN-NH2-5FU in PBS. In contrast to MSN-NH2-5FU, the zeta potential of MSN-NH2-5FU-FA was increased to 18.5 ± 0.5 at pH 5.5, which can be attributed to the increase in the ionization of the amino group of the folate. We found that MSN-NH2 could have mucoadhesive properties due to the formation of an ionic complex with the negatively charged mucin. However, the folate formulation showed a low positive charge at both pHs, meaning that it is not expected to interact with protein, as a highly charged carrier will interact strongly with macromolecules [18]. Also, folate formulation can be applied locally for oral use or by intraperitoneal administration, as we do not expect an interaction with macromolecules.

**Figure 2:**
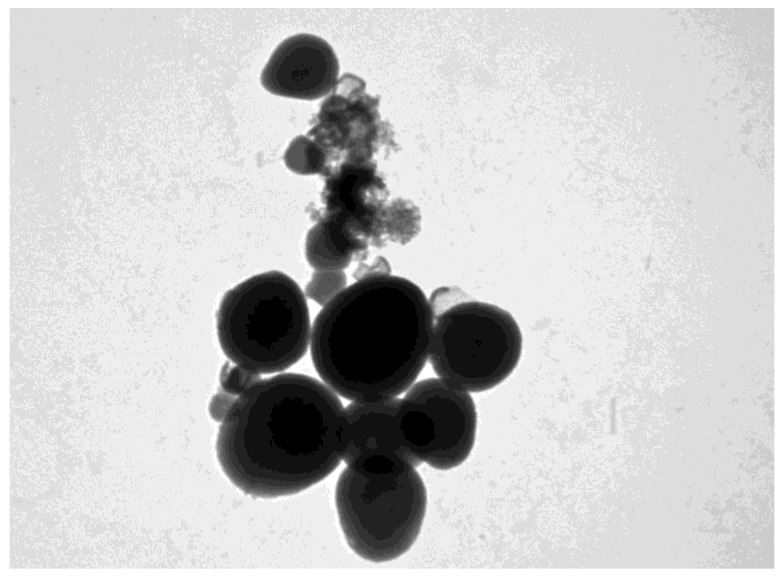
TEM images of MSN-NH2-5FU-FA.

**Figure 3:**
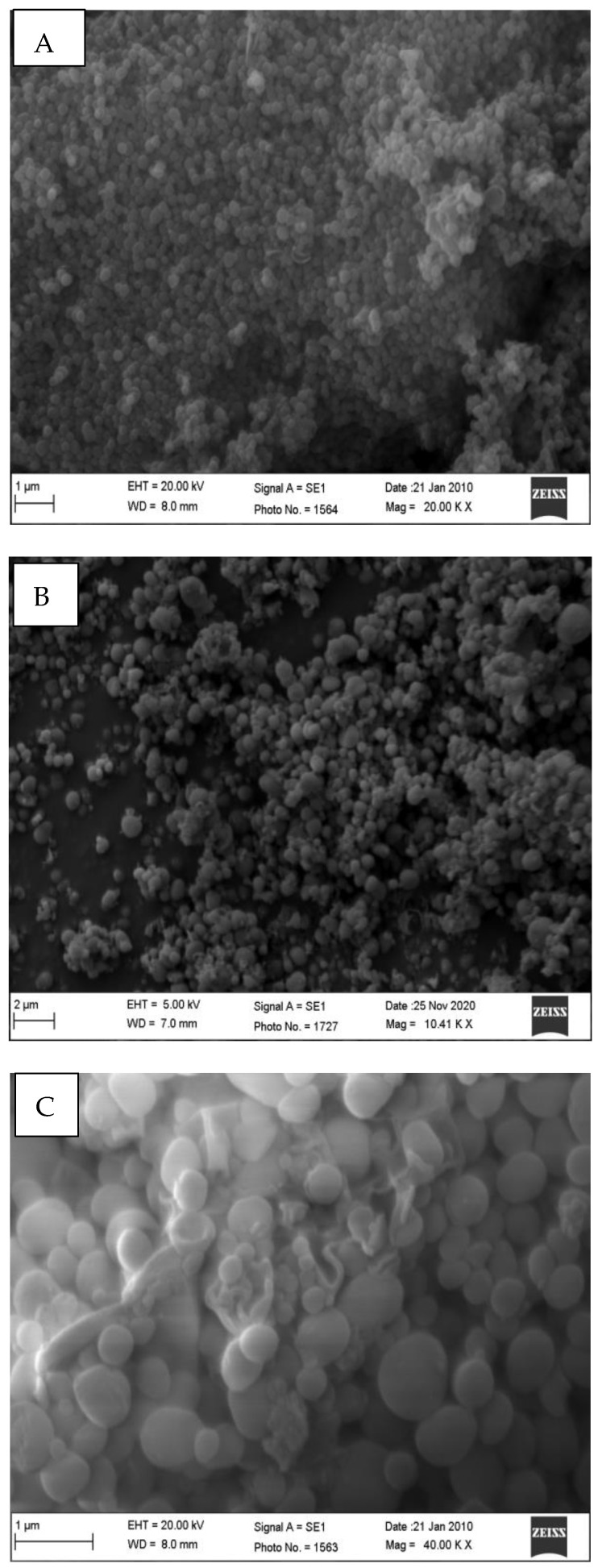
SEM images of MSN-NH2 (**A**) and MSN-NH2-5FU (**B**) MSN-NH2-5FU-FA (**C**).

**Figure 4:**
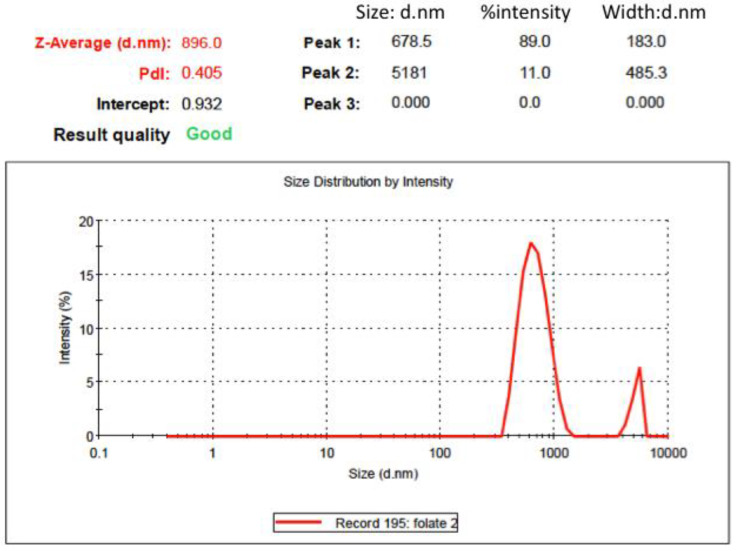
Particle size of MSN-NH2-5FU-FA by DLS.

The TEM images of the MSN-NH2-5FU-FA (Figure 2) had a fuzzy appearance and showed the low visibility of mesoporous channels, indicating that layers of folate were present on the surface of the MSN-NH2. The TEM images of MSN-NH2 and MSN-NH2-5FU were presented and described in previous work [30]. The SEM images showed that the spherical morphology of MSN-NH2-5FU (Figure 3B) was not disrupted. However, the surface of MSN-NH2-5FU-FA (Figure 3C) showed a prominent protrusion compared with MSN-NH2 and MSN-NH2-5FU (Figure 3A,B), which indicated that MSN-NH2-FA was successfully coated by the folate shell. In addition to TEM and SEM, DLS (Figure 4) showed that the MSN-NH2-5FU-FA had a larger particle size, around 907.6 ± 10.21 nm, compared to MSN-NH_2_.

### 3.3. Fourier Transform Infrared Spectroscopy (FTIR)

**Figure 5:**
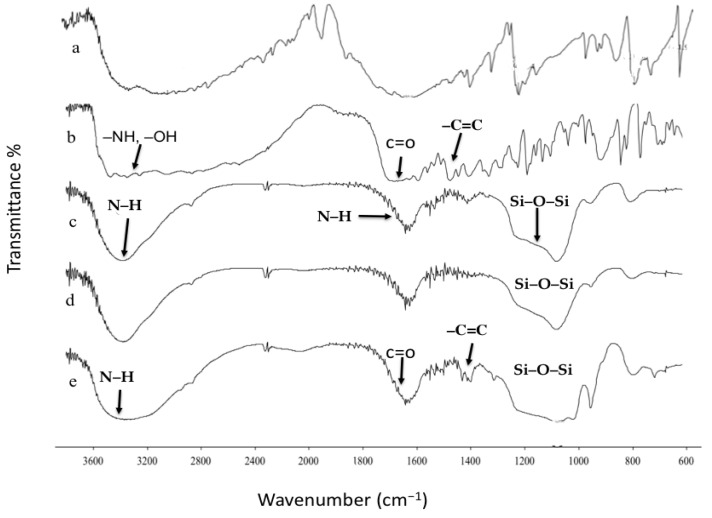
The FTIR spectra of (**a**) 5-FU, (**b**) folic acid, (**c**) MSN-NH2, (**d**) MSN-NH2-5FU, and (**e**) MSN-NH2-5FU-FA.

Figure 5a–e shows the FTIR spectra for pure 5-FU, folic acid, MSN-NH2, MSN-NH2-5FU, and MSN-NH2-5FU-FA. The fingerprint peaks of 5-FU were found at 3160, 1727, 1662, 1426, 1247, 811.7, and 547 cm^−1^ due to the vibrations of imide stretch (amide II and amide III) and the aromatic ring in the structure of the drug. Considering the folic acid chemical structure (Figure 5b), the FTIR spectrum shows a peak at the 3600–2400 cm^−1^ range, related to –NH and –OH bonds [61]. The (–C=O) group, related to pterin structure, was observed at 1694 cm^−1^. The peaks at 1635 cm^−1^ ((–C=N) stretching) and 1597 cm^−1^ belong to the bending of (–CONH_2_), and the peak at 1477 cm^−1^ is related to the (–C=C) stretching of phenyl and pterin ring [62]. Figure 5c–e shows the FTIR spectra of MSN-NH2, MSN-NH2-5FU, and MSN-NH2-5FU-FA. The strong peaks at around 1080, 950, and 800 cm^−1^ are assigned to Si–O–Si, Si-OH, and Si–O, respectively, which are specific peaks in the MSN, and the broadband at around 3400 cm^−1^ is attributed to the N–H. The band that appeared at around 1600 cm^−1^ belongs to the vibration of N–H. The band at about 2900 cm^−1^ corresponds to the C–H stretching vibration. Most of the characteristic peaks of 5-FU were not observed in the drug-loaded nanoparticles, indicating that 5-FU was encapsulated inside MSN-NH2, as previously reported (Figure 5d) [30]. Furthermore, the shifts of the peaks at about 1700 cm^−1^ of the folic acid spectrum to 1650 cm^−1^ in the MSN-NH2-5FU-FA spectrum and the overlapped peaks between 2000 and 3500 cm^−1^ in the folate spectrum, which merge in the MSN-NH2-5FU-FA spectrum, could be attributed to the formation of an amide bond after modification of the drug-loaded MSN-NH2 with folate (Figure 5e) [17,23,51,52,53,61,62]. Other folate peaks were retained in the same position in the MSN-NH2-5FU-FA spectrum, confirming the successful conjugation of the nanoparticles with folate.

### 3.4. Differential Scanning Calorimetry (DCS)

**Figure 6:**
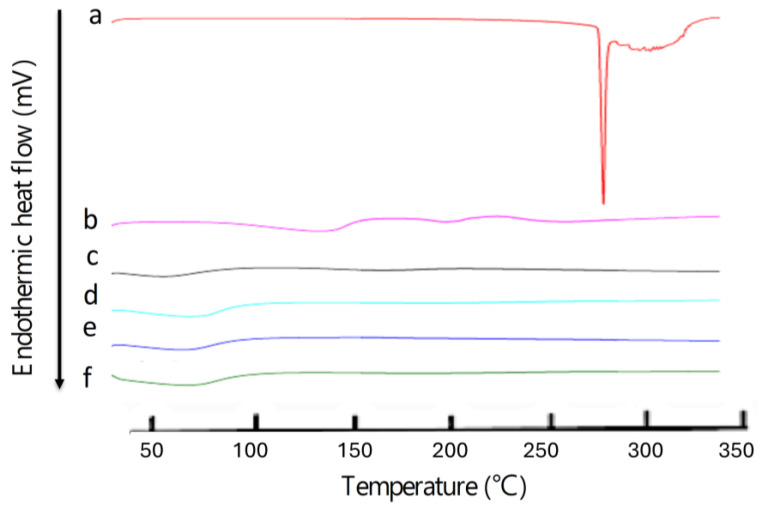
The DSC thermogram of (**a**) 5-FU, (**b**) folic acid, (**c**) MSN-NH2, (**d**) MSN-NH2-FA, (**e**) MSN-NH2-5FU, and (**f**) MSN-NH2-5FU-FA. Detailed thermograms of each sample are presented in Supplementary Materials, Figure S1.

The DSC analysis also confirmed drug entrapment into MSN-NH2, as described in previous work [30]. The thermogram of the drug in Figure 6 shows a single sharp endothermic peak at 286.76 °C (ΔH = 223.6118 J/g), corresponding to the melting event of the drug. However, the peak in the drug melting disappeared in the DSC thermograph after drug loading in the nanoparticles (MSN-NH2-5FU), suggesting that the drug was in amorphous form, and that 5-FU was successfully encapsulated inside the nanoparticle (Figure 6e). A similar scenario was found with folate formulation (MSN-NH2-5FU-FA), which showed no melting endotherm of the MSN-NH2-5FU-FA drug (Figure 6f) and confirmed the drug’s encapsulation inside the nanoparticles. The DSC curve of MSN-NH2 in Figure 6c shows two endothermic events at around 59.78 (ΔH = 80.4056 J/g) and 169 °C (ΔH = 43.2251 J/g). After the loading of 5-FU, there was only one endothermic peak at 69 °C (ΔH = 149.90 J/g), suggesting an interaction between 5-FU and nanoparticles. Functionalized MSN-NH_2_ with folic acid in MSN-NH2-FA and MSN-NH2-5FU-FA showed one endothermic peak at around 74 °C (ΔH = 155.55 J/g) and around 70 °C (ΔH = 236.70 J/g), respectively, which also suggested an interaction between the folate and the nanoparticles. Several reports showed that the folic acid exhibits some endothermic peaks at 138.0 and 201.3 °C, and then an endothermic peak for the melting of folic acid above 250 °C (Figure 6b) [63,64,65]. These studies reported that when folic acid is heated, the “Glu” moiety will first break down, followed by the degradation of pterin and PABA moieties. Afterward, when the sample was further heated, it lost its amide and acid functionalities, and the crystalline folic acid degraded at above 250 °C in the amorphous form [64]. However, the DSC thermogram of MSN-NH2-FA (Figure 6d) and MSN-NH2-5FU-FA (Figure 6f) showed no intrinsic peaks of folic acid, suggesting that the amorphous form of folate was present in the nanoparticles. Moreover, folic acid could be changed to a more heat-stable form when conjugated to the MSN-NH2, as all endothermic peaks of folic acid disappeared [64].

### 3.5. The Proton Nuclear Magnetic Resonance (^1^H-NMR)

**Figure 7:**
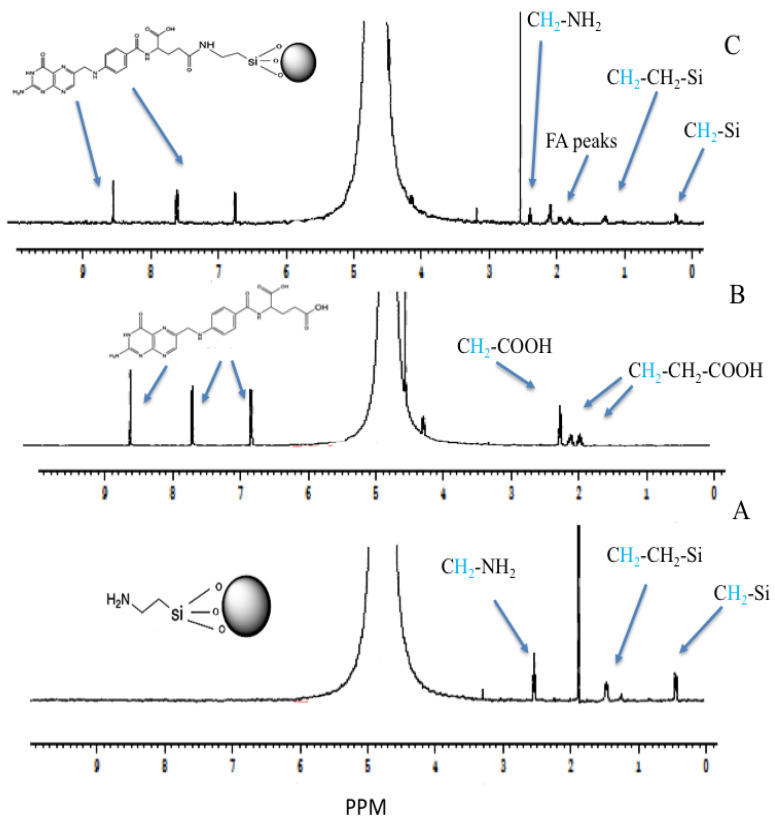
The ^1^H-NMR spectra of (**A**) MSN-NH2, (**B**) folic acid, and (**C**) MSN-NH2-FA.

The NMR spectra of MSN-NH2, folic acid, and the decorated formulation with folate (MSN-NH2-FA) are presented in Figure 7. All samples were prepared in sodium hydroxide (250 mM), in which the lyophilized samples (20 mg/mL) were solubilized and measured in D_2_O because the spectra of MSN-NH2 formulations were not evident when running the spectra directly in plain D_2_O [66]. The spectrum of MSN-NH2 was in agreement with MSN-NH2 found in the literature and measured in a sodium hydroxide solution [67,68,69,70]. The district peaks of MSN-NH2 were observed as follows: the methylene functionality of the 3-aminopropyl group appears at 2.45 triplet (CH_2_ connected to N), 1.39 quintet (middle CH_2_), and 0.35 ppm triplet (CH_2_ connected to Si). They shifted to a lower ppm due to the preparation of the samples in an alkaline media [67]. The strong peak at 1.8 ppm can be attributed to impurities in the MSN-NH2 samples. These peaks confirm the successful preparation of MSN-NH2 that was suggested in the previous work [31]. The peak values of the ^1^H-NMR spectra of folic acid were as follows: 8.5 ppm (singlet), 7.58 (doublet), and 6.7 (doublet) for aromatic-H. Peaks were observed at 4.45 [aromatic-CH_2_-NH] and 4.2 PPM for NH-CH-COOH. Peaks were observed at 2.2 and 2.05 ppm for (COOH-CH_2_-CH_2_), and peaks were observed at 1.92 ppm (COOH-CH_2_) (Figure 7B). Some folic acid spectrum peaks disappear due to proton ionization in alkaline media [71,72]. There are some new peaks in MSN-NH2-FA (Figure 7C) and, in comparison, with the MSN-NH2 spectrum (Figure 7A), attributed to FA. H-NMR spectra confirmed the successful conjugation of MSN-NH2 with folic acid. The folate moiety and MSN-NH2 peaks were retained in the MSN-NH2-FA conjugate, especially the three folate peaks of 6.5–8.5 ppm. These peaks confirmed the folate’s conjugation to MSN-NH2. The peak assigned to impurities in MSN-NH2 was slightly shifted to 2.4 ppm in MSN-NH2-FA [17,28,71,73]. The conjugation could be attributed to the formation of an amide bond after MSN-NH2 modification of folate.

### 3.6. In Vitro Drug Release

**Figure 8:**
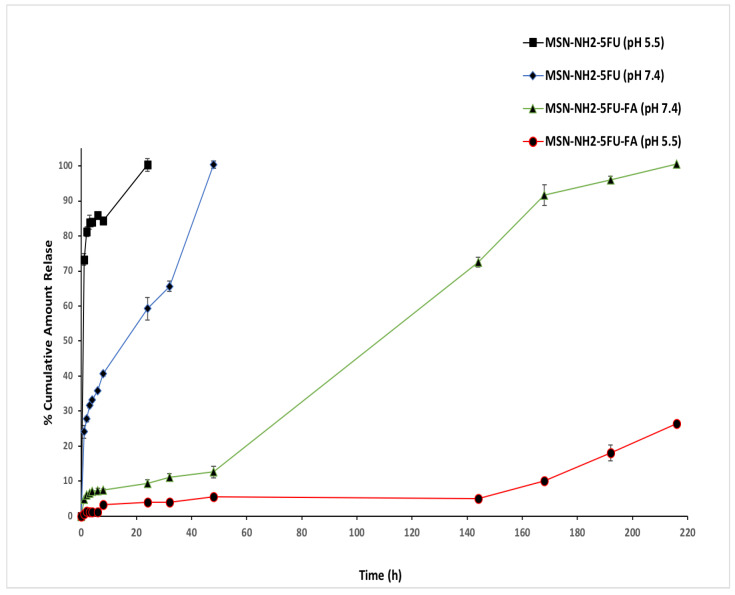
In vitro drug release profiles at pH 7.4 and 5.5 of MSN-NH2-FU and MSN-NH2-5FU-FA. Results are represented as mean ± SD (*n* = 3).

The release profile of 5-FU from MSN-NH2 in PBS pH 7.4 was slow and controlled (Figure 8). After 24 h, 59.22% of 5-FU was released from the MSN-NH2, and 100% of the drug was released in 48 h. However, the release rate of 5-FU from MSN-NH2 at pH 5.5 was faster, with a high burst release. After 6 h, around 85.87% was released, and in 24 h, about 100% of 5-FU was released from the nanoparticles. This behavior could be due to the low attraction between the MSN-NH2 and 5-FU under acidic conditions, as 5-FU becomes less ionized in acidic media, which enhances the release of 5-FU [14,74]. The release of 5-FU from MSN-NH2-5FU-FA was slower than MSN-NH2-5FU at both neutral and acidic pH, which could be due to the capping effect of folate, which decreased the porosity of MSN-NH2 by folic acid (Figure 8) [16]. After 24 h, about 9.4% was released; it took around 9 days to release 100% of 5-FU from the MSN-NH2-5FU-FA formulation at pH 7.4.

Furthermore, the release rate of the drug from MSN-NH2-5FU-FA at pH 5.5 was slower than that at pH 7.4. After 24 h, around 4% of 5-FU was released from the formulation, and after 216 h, about 26.46% of 5-FU was released. There was a difference in the surface charge at a neutral and acidic pH for MSN-NH2-5FU-FA and the slow-release profile of 5-FU at acidic media. The pKa of 5-FU is around 8, and it is a slightly soluble, weak acid drug with pH-dependent solubility (aqueous solubility around 13.56 mg/mL) [75]. Similarly, folic acid is a weak acid but is practically insoluble in water, and it showed p-dependent aqueous solubility, with higher solubility under alkaline conditions [60]. One explanation for this type of release of the MSN-NH2-5FU-FA formulation is due to the low aqueous solubility of the folate on the surface of the nanoparticles, as well as the low aqueous solubility of 5-FU under slightly acidic conditions. In contrast, at a higher pH 7.4, the solubility of 5-FU and folate increases, facilitating the release of the drug from the carrier by increasing the water uptake inside the nanoparticles’ core, creating channels for drug release. Furthermore, at pH 7.4, the MSN-NH2 surface exhibits a low positive charge and the interaction with the drug would reduce, as the drug possesses a low negative charge at this pH [38,60,74,76,77].

In the formulation of the nanoparticle system, understanding the microenvironment of cancer is significant to designing and modulating drug delivery release to achieve a high targeting efficacy and minimal leakage of the active therapeutic. A drug-loaded nanocarrier would be more effective if the drug is still loaded in the system in normal tissue with a pH of 7.4 and the carrier releases the drug at the cancer site with a lower pH of 5.5. The drug release profile from the MSN-NH2-FU formulation showed that the cumulative percentage that was released was higher at pH 5.5 than at 7.4 (Figure 8). Therefore, the MSN-NH2-FU formulation would be more stable at pH 7.4. This is significant for applications in the body in conditions where prolonged drug circulation would accumulate in cancer tumors [78]. In the release profile of MSN-NH2-FU-FA, a biphasic drug release pattern with a slow initial release followed by a sustained release was presumably attributed to the system matrix, forming a barrier to the drug release [16]. Compared with pH 5.5, the drug release was considerably faster at pH 7.4, which indicates the higher stability of MSN-NH2-5FU-FA in acidic media.

Also, the release of the drug from MSN-NH2-5FU-FA lasts around 9 days at pH 7.4, indicating good stability in normal tissues. 5-FU is used for colon cancer, along with other chemotherapeutic agents. However, 5-FU causes severe side effects after intravenous administration, and it is not administered orally due as 5-FU decomposes before reaching the colon. The efficacy of 5-FU in cancer therapy would be improved by the localized delivery of 5-FU in colon cancer. A combination of the targeting ability and the release profile of MSN-NH2-5FU-FA could be utilized for the oral route of 5-FU by preventing drug release in the upper gastrointestinal tract and facilitating its accumulation in the colonic region in a slightly basic environment, thus decreasing the systemic exposure and toxicity of the drug [54,78].

The folate receptors are overexpressed in many types of gynecological cancer cells, such as ovarian and cervical cancer [79]. Therefore, folic acid can be used as a targeting agent when treating metastatic cancers, particularly at the advanced stage. Based on this hypothesis, folic acid has been conjugated with many anticancer agents for selective targeting against cancer cells [80]. Cellular nanoparticles conjugated to folate, mainly due to the interaction of folic acid with upregulated folate receptors on the surface of cancer cells, resulting in selective cell internalization of the nanocarrier via receptor-mediated endocytosis [17,81]. Then, the endosome is trafficked to the endosomal–lysosomal compartment with an acidic pH [82]. The lysosome has some degradation enzymes that could degrade some drugs, such as 5-FU, once released in the lysosomes, and could also develop cancer resistance [82,83]. Thus, this release profile would be favorable to prevent the release of the drug in the endosomal–lysosomal compartment. The release profiles of 5-FU from the formulations at two different pH values of 7.4 and 5.5 represent the biological environment (bloodstream) and endocytic compartments, respectively [17]. Also, the positive charge of the MSN-NH2-5FU-FA at pH 5.5 would allow the nanoparticle to escape the endosomes via the proton sponge mechanism, releasing 5-FU into the cytosol and enhancing the drug efficacy [82,84]. The distinctive different release behaviors of MSN-NH2-FU and MSN-NH2-FU-FA indicated that drugs encapsulated in FA-conjugated nanoparticles could release 5-FU as a sustained type of formulation for a longer time. The delayed release of the drug from MSN-NH2-FU-FA indicates that it can act as an efficient drug carrier and could minimize the exposure of anticancer agents to normal tissues and enhance the accumulation in tumor tissues [37].

All the release data were fitted by zero-order, first-order, Higuchi kinetic, and Korsmeyer–Peppas Hixen–Crowell kinetic models Table 3. MSN-NH2-5FU, best described by Higuchi, is the type of release best described as R^2^ and is the highest, with a value of 0.95. The Korsmeyer–Peppas plot determines the detailed drug release mechanism from the system. The Korsmeyer–Peppas plot logarithm of the cumulative percentage drug release over the logarithm of time indicated good linearity at pH 7.4 (R^2^ = 0.98) for MSN-NH2-5FU, with an *n* value equal to 0.3. The obtained *n* value was less than 0.5, which suggests that Fickian diffusion release profiles occur at pH 7.4 for the MSN-NH2-5FU formulation. Plotted data indicate that the highest R^2^ agreed with the Korsmeyer–Peppas and Higuchi models, suggesting that the drug release from MSN-NH2-5FU occurred via diffusion and relaxation [85]. Also, it was shown that the drug release from MSN-NH2-5FU-FA at pH 7.4 was best described by the zero-order model. The release profile of MSN-NH2-5FU-FA showed the highest linearity, with an R^2^ of 0.98, when fitted to the zero-order release model. The release profile was not applicable to the drug release experiment at pH 5.5 for MSN-NH2-5FU because the amount of the initial cumulative drug released after 1 h was higher than 60%, and the drug release data do not fit any models with good linearity. Also, the MSN-NH2-5FU-FA release profile is not suitable, as the cumulative amount released in the experiment was insufficient to fit any drug’s release model. The Higuchi and zero-order models are two limit cases in the drug transport mechanism [55]. In polymeric matrix systems, the Higuchi model is widely used to compare two drug dissolution profiles. When describing coated dosage forms or membrane control, such as the MSN-NH2-5FU-FA, the zero-order model works best [55]. Thus, the drug release from MSN-NH2-5FU-FA could occur through a mechanism such as a drug diffusion along with the erosion of the folate layer that forms the membrane on silica nanoparticles, resembling the nanocapsules’ release profile.

### 3.7. Ex Vivo Drug Permeation Experiment

**Figure 9:**
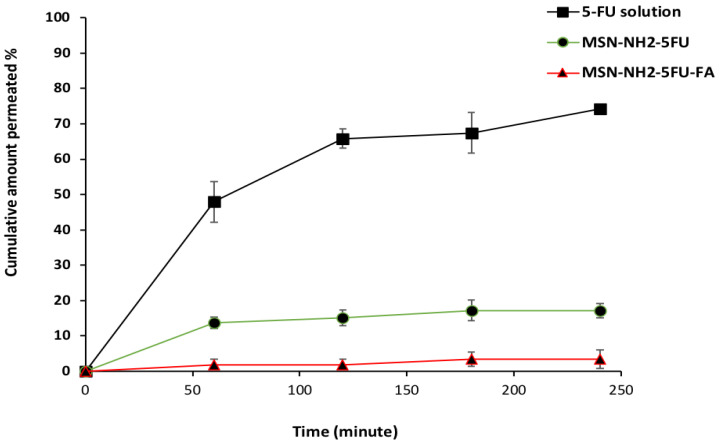
Ex vivo intestinal permeability profiles of MSN-NH2-5FU-FU, MSN-NH2-5FU-FA, and 5-FU drug solution. Results are represented as mean ± SD (*n* = 3).

An ex vivo permeability study indicated significantly higher apparent permeability (Papp) for the 5-FU solution compared to MSN-NH2-5FU and MSN-NH2-5FU-FA-FU-FA (Figure 9). The Papp for 5-FU solution, MSN-NH2-5FU, and MSN-NH2-5FU-FA was found to be 13.2 × 10^−5^ cm/s ± 9.37 × 10^−6^, 6.15 × 10^−5^ cm/s ± 3.54 × 10^−6^, and 1.48 × 10^−5^ cm/s ± 4.93 × 10^−6^, respectively. Also, the cumulative amount permeated from the 5-FU solution was 74.32 ± 0.5% and permeated across the intestinal membrane after 4 h, which was significantly higher than those of MSN-NH2-5FU and MSN-NH2-5FU-FA, which were 17.16 ± 2.09% and 3.36 ± 2.64%, respectively, at the same time point. The cumulative amount that permeated and the Papp of MSN-NH2-5FU-FU was significantly higher (*p* < 0.01) than those of MSN-NH2-5FU-FA. Also, a high Papp was obtained for the 5-FU solution in comparison to MSN-NH2-5FU and MSN-NH2-5FU-FA because the drug can pass the intestinal membrane at a high rate and amount [86,87]. The low Papp and cumulative amount permeated % in MSN-NH2-5FU-FA suggest that permeation of the nanoparticle can be inhibited by coating the particle with folate molecules that can be utilized for the delivery of MSN-NH2-5FU-FA to the colon region and for targeting the folate receptors in colorectal cancer [17]. The low permeability of the MSN-NH2-5FU formulation in comparison to the drug solution could be due to the limited time of the intestinal permeation experiment, which cannot be extended for a longer time, which causes necrosis of the intestinal tissues and does not reflect the actual permeation of the MSN-NH2, which needs a longer time [88,89].

### 3.8. Cell Viability Study in Ovarian and Cervical Cancer Cells

**Figure 10:**
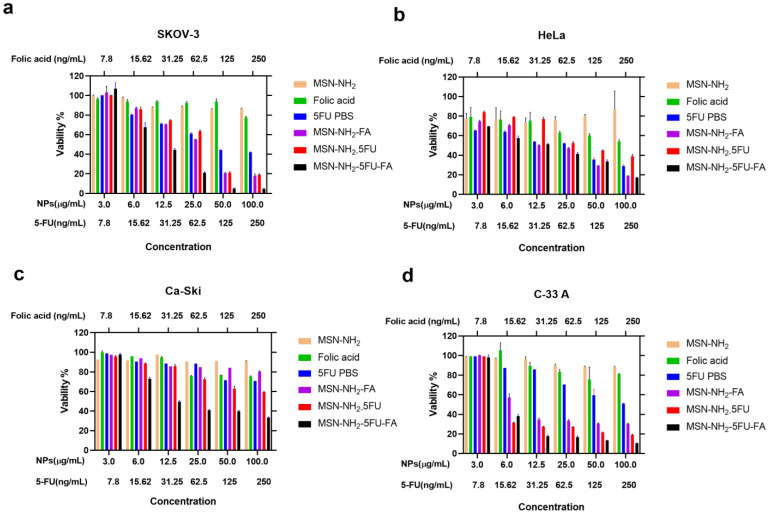
Cytotoxicity in SKOV-3 (**a**), HeLa (**b**), Ca-Ski (**c**), and C-33 A (**d**) cells after 72 h or treatment. Data are represented as mean ± SD (*n* = 3). Statistical significance was obtained with *p*-values ≤ 0.05. Statistical significance can be found in Supplementary Materials, Tables S1–S4.

The MTT assay is in agreement with our previous published study, where drug-free MSN-NH2 showed no toxicity to all cells, which might be attributed to the main surface functionalization, which effectively mitigates the interaction between silanol moieties on the surface of the nanoparticles and cells, thereby reducing their potential toxicity [90]. On the other hand, MSN-NH2-5FU-FA exerted a significant difference (67%) in viability in SKOV-3 cells compared to free 5-FU, which was apparent at 15.625 ng/mL of 5-FU as well as six µg/mL MSN-NH2, MSN-NH2-5FU, and MSN-NH2-FA, in comparison to the other formulations (cells viabilities were 80%, 97%, 85%, and 85.9%, respectively) (Figure 10a).

Although initially, HeLa cells showed a decrease in cell viability at a 5-FU concentration of 7.8 ng/mL, which was lower than but not significantly different to MSN-NH2-5FU-FA, a significant difference began to appear at 15.625 ng/mL of 5-FU and six µg/mL of MSN-NH2-5FU-FA in a similar scenario to SKOV-3 (Figure 10b). Caski cell viability was also significantly decreased by MSN-NH2-5FU-FA compared to the other treatment group (Figure 10c). However, cell viability did not reach less than 33% with the highest 5-FU and MSN-NH2-5FU-FA concentrations used in this study. It seems that C-33A cells were the most responsive cervical cancer cells to MSN-NH2-5FU-FA treatment, reaching only 17% cell viability at 12.5 µg/mL MSN-NH2-5FU-FA, while free 5-FU exhibited 87% cell viability. Furthermore, C-33 A cells showed a significant decrease in cell viability with MSN-NH2-5FU and MSN-NH2-FA compared to free 5-FU and FA. Previous reports indicated that folate can inhibit C-33 A cell proliferation [91]. This might indicate that the cytotoxic effect found with C-33 A cells (Figure 10d) may be attributed to a synergist effect due to the presence of both 5-FU and FA in the MSN-NH2-5FU-FA. Furthermore, it seems that HPV-negative cervical cancer cells are more sensitive than HPV-positive cells toward 5-FU therapy.

In general, the higher cytotoxic effect seen with MSN-NH2-5FU-FA could be attributed to the presence of active receptors that facilitate the endocytosis of the amino-functionalized silica nanoparticles and the controlled release of the drug from the silica [92,93].

### 3.9. Cellular Uptake Study

**Figure 11:**
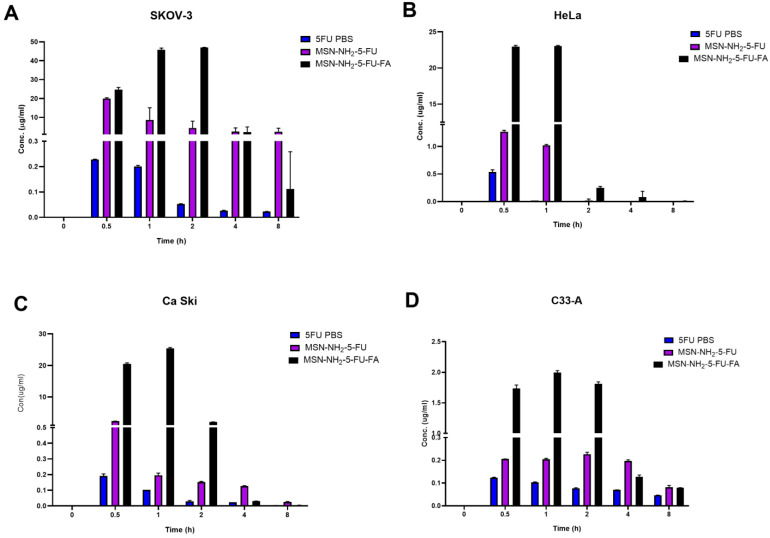
Cellular uptake study in SKOV-3 (**A**), HeLa (**B**), Ca-Ski (**C**), and C-33 A (**D**) cells measured after cell treatment with MSN-NH2-5FU-FA, MSN-NH2-5FU, and free 5-FU. Data are represented as mean ± SD (*n* = 3). Statistical significance was obtained with *p*-values ≤ 0.05. Statistical significance can be found in Supplementary Materials, Tables S5–S8.

**Figure 12:**
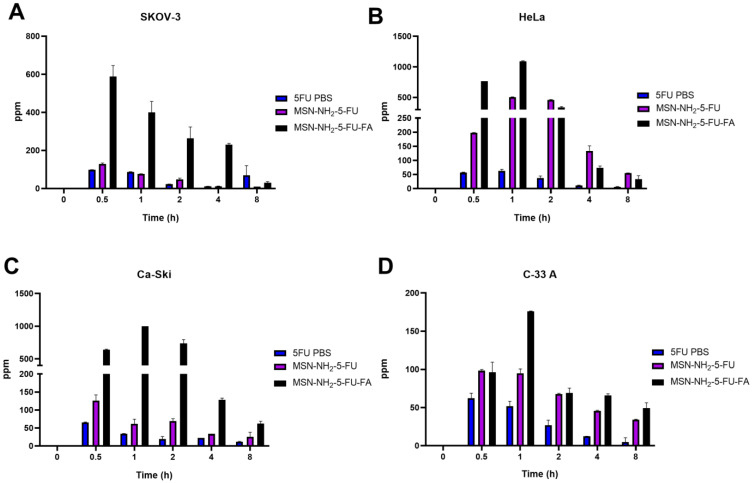
The cellular uptake of nanoparticles SKOV-3 (**A**), HeLa (**B**), Ca-Ski (**C**), and C-33 (**D**) cells was measured using ICP-MS with ^51^Sb as an internal standard. The data are presented as the mean ± SD (*n* = 3). Statistical significance was obtained with *p*-values ≤ 0.05. Statistical significance can be found in Supplementary Materials, Tables S9–S12.

To evaluate the cellular uptake potential of nanoparticles, a non-direct method was used to quantify the amount of 5-FU in cells after treatment with the free drugs MSN-NH2-5FU and MSN-NH2-5FU-FA. An increase in 5-FU concentration was apparent after 0.5 h of treatment and up to 2 h, with a significant difference in 5-FU concentration in MSN-NH2-5FU-FA compared to free and MSN-NH2-5FU-treated cells (Figure 11). These results were confirmed by quantifying the amount of 5-FU uptake by cells from the 5-FU solution compared to MSN-NH2-5FU and MSN-NH2-5FU-FA using ICP-MS (Figure 12). The results showed a significant difference between 5-FU levels in the MSN-NH2-5FU-FA-treated group compared to free 5-FU and MSN-NH2-5FU in SKOV-3 cells. However, there was no significant difference in 5-FU between the free 5-FU and MSN-NH2-5FU-treated cells. Although there was a significant difference in 5-FU concentration between the free 5-FU-treated cells and both MSN-NH2-5FU- and MSN-NH2-5FU-FA-treated HeLa and Ca-Ski cells indicating an enhancement of drug delivery, the amount of 5-FU quantified in the MSN-NH2-5FU-FA was significantly higher than that in the MSN-NH2-5FU groups. A similar scenario was found with C-33 A cells. However, the significance between the 5-FU concentration between the MSN-NH2-5FU and MSN-NH2-5FU-FA groups was apparent 1 h after treatment compared to the 30 min observed with another type of cells. The intracellular uptake results were consistent with the MTT assay results. As mentioned previously, the high 5-FU concentration after MSN-NH2-5FU-FA could be due to the increase in NP uptake by the folate receptors [92,93]. However, further studies are needed to confirm the safety and efficacy profiles in vivo.

## 4. Conclusions

This work highlighted a simple method for the preparation and characterization of MSN-NH nanoparticles with folate surface conjugation that would enhance the activity of 5-FU. This simple procedure retains the superior activity of the folate formulation in comparison to non-folate-conjugated nanoparticles and the free drug solution. Structural elucidation by NMR and FTIR confirms the functionalization of MSN-NH2 with folate moiety. Also, DSC analysis further illustrates the physical state of the drug and the carrier. Moreover, the prepared formulation showed a sustained release profile with a pH-dependent release that can be further optimized for intraperitoneal administration for gynecological cancer. The pH-dependent release would prevent the release of the drug under low-pH conditions, which could reduce the degradation of 5-FU in the lysosomes and hence enhance the activity of the drug. The folate-functionalized formulation showed the pH-dependent and controlled release of the drug from nanoparticles. This slow-release profile of 5-FU from the nanoparticle was suggested to be beneficial in extending the drug activity and reducing drug resistance and toxicity [94,95]. Also, the pH-dependent release would enhance the localization of the drug in the cancer, as in the case of colorectal cancer, as the small intestine and colon have slightly alkaline conditions [19,96,97,98]. It would also prevent the degradation of 5-FU in low-pH conditions such as the cancer microenvironment and lysosomal compartment by preventing the premature release of the drugs [84]. Some reports suggested that positive-charge particles improve the cellular particle uptake and further enhance the lysosomal escape of the drug [84,93]. The encapsulation of 5-FU in MSN-NH2 and functionalization with folate would enhance the overall performance of the delivery of 5-FU in comparison to the unconjugated nanoparticles. The observed anticancer activity of folate formulation presents a possibility for its use in malignancy treatments, such as treatment for ovarian and cervical cancers, encouraging further investigation in an in vivo model. Thus, MSN-NH2-FA could be a potential carrier for 5-FU in the treatment of gynecological cancers.

## Figures and Tables

**Table 1:** Advantages in disease treatment achieved via the use of some nanoparticulate systems for 5-FU delivery.

System	Cancer	Advantages	Reference
Solid lipid nanoparticles	Colorectal	Enhanced anticancer activity	[32]
Chitosan nanoparticles	Colorectal	Better targeting efficiency and localized drug in cancer cells	[33]
PLGA nanoparticles	Colorectal	Maximum cell-lysis effect and better targeting efficiency	[34,35]
Citrus pectin nanoparticles coated with Eudragit S100	Colorectal	Prolonged drug release and enhance selectivity	[36]
Silica nanoparticles conjugated to hyalurocin acid	Colon	Enhanced cellular uptake and improved cytotoxicity	[37]
Aminofunctionalized MSN	Colorectal	Improved cytotoxicity and pH-responsive controlled drug release system	[38]
Alginate–chitosan nanoparticles	Ocular application	Enhanced ocular absorption and pharmacokinetics	[39]
Galactosylated chitosan functionalized MSN	Colon cancer	Enhanced anti-cancer activity and targeting	[16]
Chitosan and PEG coated MSNs	Breast and cervical cancer	Enhanced anti-cancer activity with the loading of two anticancer drugs	[19]
MSN	Melanoma	Enhanced anti-cancer activity and skin permeation	[30]
Carboxymethyl chitosan-coated MSN	Ocular application	Enhance ocular absorption and pharmacokinetic	[31]
Combination of 5-FU and cisplatin with electroporation	Ovarian	Enhanced anti-cancer activity	[40]
Folic acid and PLGA conjugates	Colorectal	Enhanced anticancer activity	[41]
Folate-conjugated polymers	Colon	Enhanced anti-cancer activity and targeting	[42]
PLGA folate-conjugated peptide nanoparticles	Melanoma	Enhanced cytotoxicity and targeting	[43]
Bi-MIL-88B MOF nanoparticles coated with chitosan–folic acid conjugate	Colon	Enhanced anti-cancer activity and targeting	[44]
MSN nanoparticles coated with folic acid-modified lipid	Breast cancer	Enhanced cytotoxicity and targeting with the loading of two anticancer drugs	[45]

**Table 2:** Particle size, polydispersity index, zeta potential, EE%, and LC% of MSN-NH2, MSN-NH2-5FU, and MSN-NH2-5-FU-FA (mean ± SD).

	MSN-NH2	MSN-NH2-5FU	MSN-NH2-5FU-FA
Particle size (nm)	169.3 ± 4.2	193.9 ± 8.7	907.6 ± 10.21
Polydispersity index (PDI)	0.057 ± 0.002	0.219	0.405 ± 0.001
Zeta potential (mV) in pH 5.5	27.1 ± 0.75	18 ± 2.9	18.5 ± 0.5
Zeta potential (mV) in PBS	6.36 ± 1.49	30.4 ± 5.71	8.57 ± 3.48
EE%	-	18.01 ± 3.7	13.05 ± 0.73
LC%	-	15.26 ± 3.13	5.89 ± 0.32

**Table 3:** Regression values of different release kinetic models.

Formulation	pH	Zero-Order (R^2^)	First Order (R^2^)	Higuchi (R^2^)		Korsmyer–Peppas (*n*)	Hixen–Crowell (R^2^)
MSN-NH2-5FU	7.4	0.904	0.77	0.95	0.98	0.3	0.79
MSN-NH2-5FU-FA	7.4	0.98	0.91	0.91	0.85	0.413	0.73

## Data Availability

All data are represented within the manuscript or in Supplementary Materials.

## Supplementary Materials

The following supporting information can be downloaded at: https://www.mdpi.com/article/10.3390/jfb15030074/s1, Figure S1: DSC thermograms of (a) 5-FU, (b) folic acid, (c) MSN-NH2, (d) MSN-NH2-FA, (e) MSN-NH2-5FU, and (f) MSN-NH2-5FU-FA. Tables S1–S4: Statistical significance in cell viability between different treatment groups in SKOV-3, HeLa, Ca Ski, and C-33 A cells. Tables S5–S8: Statistical significance in cellular uptake between different treatment groups in SKOV-3, HeLa, Ca Ski, and C-33 A cells. Tables S9–S12: Statistical significance of the cellular uptake of nanoparticles SKOV-3, HeLa, Ca Ski, and C-33 cells was measured using ICP-MS with ^51^Sb as an internal standard.
