# Supplementary material for: A Comprehensive Study on Folate-Targeted Mesoporous Silica Nanoparticles Loaded with 5-Fluorouracil for the Enhanced Treatment of Gynecological Cancers
Source: J Funct Biomater. 2024 Mar 20;15(3):74. doi: 10.3390/jfb15030074 (PMC10970946; doi:10.3390/jfb15030074)
Supplement: Supplementary file 1 [file jfb-15-00074-s001.zip › jfb-2891501-supplementary materials final.pdf]

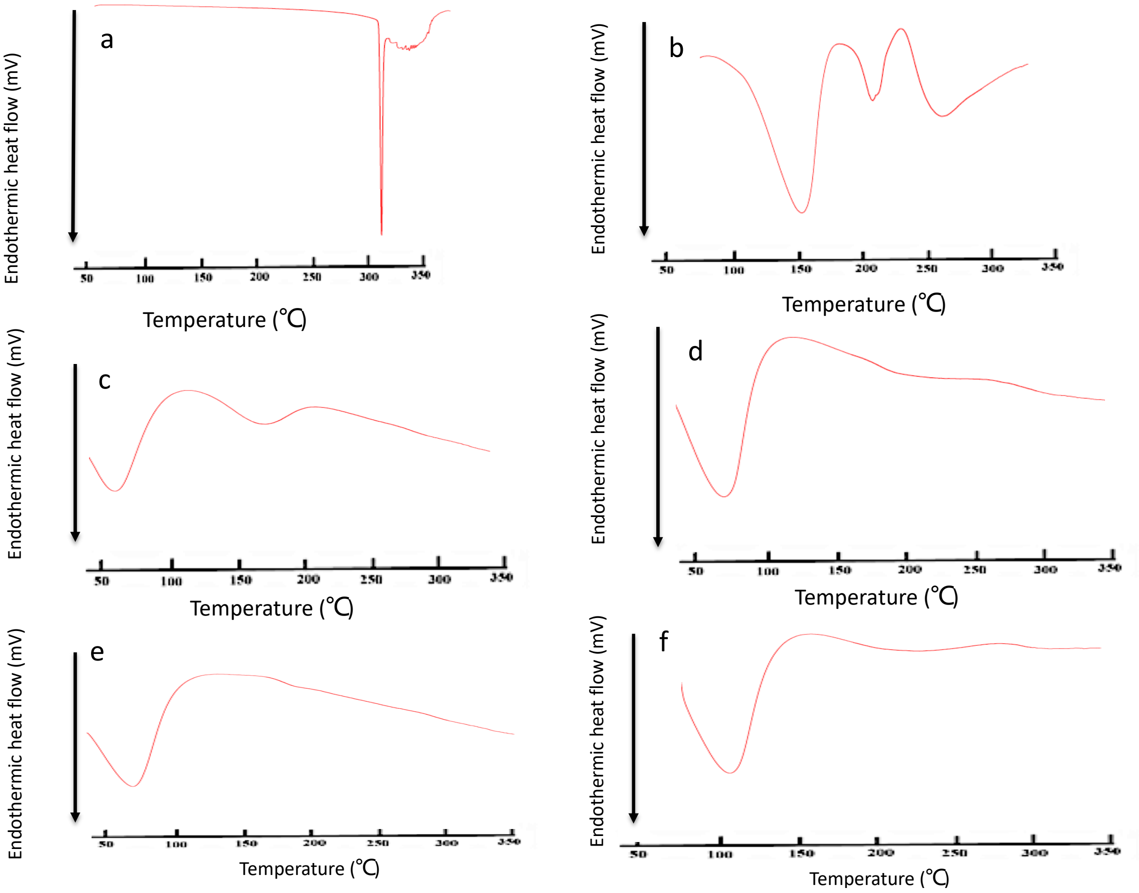

Figure S1: A) The DSC thermograms of (a) 5-FU (b) folic acid (c) MSN-NH_2 (d) MSN-NH_2-FA (e) MSN-NH_2-5FU and (f) MSN-NH_2-5FU-FA B).

Table S1. Statistical significance in cell viability between different treatment groups in SKOV-3

Statistical significance was obtained with p-values ≤ 0.05, where * p ≤ 0.05, ** p ≤ 0.01, *** p ≤ 0.001, and **** p < 0.0001.

	3.12μg/ ml	6.25 μg/ml	12.5 μg/ml	25 μg/ml	50 μg/ml	100 μg/ml
Folic acid vs. MSN-NH2-FA	Ns	*	****	****	****	****
Folic acid vs. MSN-NH2	ns	ns	****	**	****	****
Folic acid vs. MSN-NH2-5-FU	ns	*	****	****	****	****
Folic acid vs. MSN-NH2-5FU-FA	*	****	****	****	****	****
Folic acid vs. 5FU	ns	***	****	****	****	****
MSN-NH2-FA vs. MSN-NH2	ns	**	****	****	****	****
MSN-NH2-FAvs. MSN-NH2-5FU	ns	ns	***	****	ns	ns
MSN-NH2-FA vs. MSN-NH2-5FU-FA	ns	****	****	****	****	****
MSN-NH2-FA vs. 5FU	ns	*	ns	****	****	****
MSN-NH2 vs. MSN-NH2-5FU	ns	***	****	****	****	****
MSN-NH2 vs. . MSN-NH2-5FU-FA	ns	****	****	****	****	****
MSN-NH2 vs. 5FU	ns	****	****	****	****	****
MSN-NH2-5FU vs. MSN-NH2-5FU-FA	ns	****	****	****	****	****
MSN-NH2-5FU vs. 5FU	ns	ns	**	ns	****	****
MSN-NH2-5FU-FA vs. 5FU	ns	***	****	****	****	****

Table S2. Statistical significance in cell viability between different treatment groups in HeLa cells.

	3.125μg/ ml	6.25 μg/ml	12.5 μg/ml	25 μg/ml	50 μg/ml	100 μg/m
Folic acid vs. MSN-NH2-FA	ns	ns	****	****	****	**
Folic acid vs. MSN-NH2	ns	ns	ns	****	****	**
Folic acid vs. MSN-NH2-5-FU	ns	ns	ns	***	****	ns
Folic acid vs. MSN-NH2-5FU-FA	ns	*	***	****	****	***
Folic acid vs. 5FU	*	ns	***	****	****	*
MSN-NH2-FA vs. MSN-NH2	ns	ns	***	****	****	****
MSN-NH2-FAvs. MSN-NH2-5-FU	ns	ns	****	*	****	ns
MSN-NH2-FA vs. MSN-NH2-5FU-FA	ns	ns	ns	*	*	ns
MSN-NH2-FA vs. 5FU	ns	ns	ns	ns	**	ns
MSN-NH2 vs. MSN-NH2-5-FU	ns	ns	ns	****	****	****
MSN-NH2 vs. . MSN-NH2-5FU-FA	ns	*	***	****	****	****
MSN-NH2 vs. 5FU	ns	ns	***	****	****	****
MSN-NH2-5FU vs. MSN-NH2-5FU-FA	*	**	****	****	****	*
MSN-NH2-5FU vs. 5FU	**	ns	***	ns	****	ns
MSN-NH2-5FU-FA vs. 5FU	ns	ns	ns	***	ns	ns

Statistical significance was obtained with p-values ≤ 0.05, where * p ≤ 0.05, ** p ≤ 0.01, *** p ≤ 0.001, and **** p < 0.0001.

‘

Table S3. Statistical significance in cell viability between different treatment groups in Ca-Ski cells.

	3.125µg/ ml	6.25 µg/ml	12.5 µg/ml	25 µg/ml	50 µg/ml	100 µg/m
Folic acid vs. MSN-NH2-FA	*	*	****	****	****	****
Folic acid vs. MSN-NH2	****	****	*	****	****	****
Folic acid vs. MSN-NH2-5-FU	***	****	****	**	****	****
Folic acid vs. MSN-NH2-5FU-FA	ns	****	****	****	****	****
Folic acid vs. 5FU	ns	****	****	****	***	***
MSN-NH2-FA vs. MSN-NH2	***	*	****	****	****	****
MSN-NH2-FAvs. MSN-NH2-5-FU	ns	****	ns	****	****	****
MSN-NH2-FA vs. MSN-NH2-5FU-FA	ns	****	****	****	****	****
MSN-NH2-FA vs. 5FU	ns	***	*	***	****	****
MSN-NH2 vs. MSN-NH2-5-FU	**	***	****	****	****	****
MSN-NH2 vs. . MSN-NH2-5FU-FA	****	****	****	****	****	****
MSN-NH2 vs. 5FU	****	ns	****	**	****	****
MSN-NH2-5FU vs. MSN-NH2-5FU-FA	ns	****	****	****	****	****
MSN-NH2-5FU vs. 5FU	**	*	*	****	****	****
MSN-NH2-5FU-FA vs. 5FU	ns	****	****	****	****	****

Statistical significance was obtained with p-values ≤ 0.05, where * p ≤ 0.05, ** p ≤ 0.01, *** p ≤ 0.001, and **** p < 0.0001.

Table S4. Statistical significance in cell viability between different treatment groups in C-33 A cells.

	3.125µg/ ml	6.25 µg/ml	12.5 µg/ml	25 µg/ml	50 µg/ml	100 µg/m
Folic acid vs. MSN-NH2-FA	ns	****	****	****	****	****
Folic acid vs. MSN-NH2	ns	ns	**	***	ns	****
Folic acid vs. MSN-NH2-5-FU	ns	****	****	****	****	****
Folic acid vs. MSN-NH2-5FU-FA	ns	****	****	****	****	****
Folic acid vs. 5FU	ns	***	ns	****	*	***
MSN-NH2-FA vs. MSN-NH2	ns	****	****	****	****	****
MSN-NH2-FAvs. MSN-NH2-5-FU	ns	****	**	***	ns	****
MSN-NH2-FA vs. MSN-NH2-5FU-FA	ns	***	****	****	*	****
MSN-NH2-FA vs. 5FU	ns	****	****	****	***	****
MSN-NH2 vs. MSN-NH2-5-FU	ns	****	****	****	****	****
MSN-NH2 vs. MSN-NH2-5FU-FA	ns	****	****	****	****	****
MSN-NH2 vs. 5FU	ns	ns	***	****	***	****
MSN-NH2-5FU vs. MSN-NH2-5FU-FA	ns	ns	***	****	ns	****
MSN-NH2-5FU vs. 5FU	ns	****	****	****	****	****
MSN-NH2-5FU-FA vs. 5FU	ns	****	****	****	****	****

Statistical significance was obtained with p-values ≤ 0.05, where * p ≤ 0.05, ** p ≤ 0.01, *** p ≤ 0.001, and **** p < 0.0001.

‘

Table S5. Statistical significance of Intracellular uptake of 5-FU using HPLC between different treatment groups in SKOV-3cells

	0.5 h	1 h	2 h	4 h	8 h
MSN-NH2-5FU vs. 5FU	***	ns	ns	ns	ns
MSN-NH2-5FU-FA vs. 5FU	****	****	****	ns	ns
MSN-NH2-5FU vs. MSN-NH2-5FU-FA	***	****	****	ns	ns

Statistical significance was obtained with p-values ≤ 0.05, where * p ≤ 0.05, ** p ≤ 0.01, *** p ≤ 0.001, and **** p < 0.0001.

Table S6. Statistical significance of Intracellular uptake of 5-FU using HPLC between different treatment groups in HeLa cells

	0.5 h	1 h	2 h	4 h	8 h
MSN-NH2-5FU vs. 5FU	***	****	ns	ns	ns
MSN-NH2-5FU-FA vs. 5FU	****	****	****	ns	****
MSN-NH2-5FU vs. MSN-NH2-5FU-FA	****	****	****	ns	****

Statistical significance was obtained with p-values ≤ 0.05, where * p ≤ 0.05, ** p ≤ 0.01, *** p ≤ 0.001, and **** p < 0.0001.

Table S7. Statistical significance of Intracellular uptake of 5-FU using HPLC between different treatment groups in Ca-Ski cells

	0.5 h	1 h	2 h	4 h	8 h
MSN-NH2-5FU vs. 5FU	****	ns	****	****	****
MSN-NH2-5FU-FA vs. 5FU	****	****	****	**	ns
MSN-NH2-5FU vs. MSN-NH2-5FU-FA	****	****	****	****	****

Statistical significance was obtained with p-values ≤ 0.05, where * p ≤ 0.05, ** p ≤ 0.01, *** p ≤ 0.001, and **** p < 0.0001.

Table S8. Statistical significance of Intracellular uptake of 5-FU using HPLC between different treatment groups in C-33 A cells

	0.5 h	1 h	2 h	4 h	8 h
MSN-NH2-5FU vs. 5FU	*	ns	***	****	***
MSN-NH2-5FU-FA vs. 5FU	****	****	****	****	***
MSN-NH2-5FU vs. MSN-NH2-5FU-FA	****	****	****	****	ns

Statistical significance was obtained with p-values ≤ 0.05, where * p ≤ 0.05, ** p ≤ 0.01, *** p ≤ 0.001, and **** p < 0.0001.

'

Table S9. Statistical significance of Intracellular uptake of 5-FU using ICP-MS between different treatment groups in SKOV-3

	0.5 h	1 h	2 h	4 h	8 h
MSN-NH2-5FU vs. MSN-NH2-5FU-FA	****	****	***	****	ns
5FU PBS vs. MSN-NH_2-5-FU	ns	ns	ns	ns	ns
5FU PBS vs. MSN-NH2-5FU-FA	****	****	***	****	ns

Statistical significance was obtained with p-values ≤ 0.05, where * $p \leq 0.05$, ** $p \leq 0.01$, *** $p \leq 0.001$, and **** $p < 0.0001$.

Table S10. Statistical significance of Intracellular uptake using ICP-MS between different treatment groups in HeLa

	0.5 h	1 h	2 h	4 h	8 h
MSN-NH2-5FU vs. MSN-NH2-5FU-FA	****	****	****	**	*
5FU PBS vs. MSN-NH_2-5-FU	****	****	****	****	***
5FU PBS vs. MSN-NH2-5FU-FA	****	****	****	**	**

Statistical significance was obtained with p-values ≤ 0.05, where * $p \leq 0.05$, ** $p \leq 0.01$, *** $p \leq 0.001$, and **** $p < 0.0001$.

Table S11. Statistical significance of Intracellular uptake using ICP-MS between different treatment groups in Ca-Ski

	0.5 h	1 h	2 h	4 h	8 h
MSN-NH2-5FU vs. MSN-NH2-5FU-FA	****	****	****	****	**
5FU PBS vs. MSN-NH_2-5-FU	****	**	ns	**	ns
5FU PBS vs. MSN-NH2-5FU-FA	****	****	****	****	***

Statistical significance was obtained with p-values ≤ 0.05, where * $p \leq 0.05$, ** $p \leq 0.01$, *** $p \leq 0.001$, and **** $p < 0.0001$.

Table S12. Statistical significance of Intracellular uptake using ICP-MS between different treatment groups in C-33 A

	0.5 h	1 h	2 h	4 h	8 h
MSN-NH2-5FU vs. MSN-NH2-5FU-FA	****	****	****	****	**
MSN-NH2-5FU vs. 5FU PBS	****	**	ns	**	ns
MSN-NH2-5FU-FA vs. 5FU PBS	****	****	****	****	***

Statistical significance was obtained with p-values ≤ 0.05, where * $p \leq 0.05$, ** $p \leq 0.01$, *** $p \leq 0.001$, and **** $p < 0.0001$.
